# Proposing immersive virtual reality scenarios for validating verbal content analysis methods in adult samples

**DOI:** 10.3389/fpsyg.2024.1352091

**Published:** 2024-02-19

**Authors:** Judith A. Iffland, Theres Volz, Silvia Gubi-Kelm

**Affiliations:** Department Psychology, Institute for Forensic Psychology and Medicine (IFPM), Medical School Hamburg, Hamburg, Germany

**Keywords:** Criteria-Based Content Analysis, validity, statement credibility, virtual reality, verbal content analysis, lie detection, memory

## Abstract

Verbal content analyses to differentiate truthful and fabricated statements, such as the Criteria-Based Content Analysis (CBCA), are used in lie detection research as well as in practice to assess the credibility of statements in criminal court proceedings. Meta-analyses demonstrate validity of verbal content analyses above chance, but the traditional research paradigms usually lack either ecological or internal validity. The authors discuss the usage of immersive virtual reality scenarios to solve this dilemma, as both types of validity can be increased by this approach. In this integrative review of existing literature on the current use of virtual scenarios in forensic and victimology research, the authors extract strengths and limitations for possible VR studies in the context of verbal content analysis. Furthermore, novel ethical challenges involved are summarized and implications for future studies proposed. Overall, we argue in favor of using virtual reality scenarios to validate methods for verbal content analysis, but also urge to consider ethical limitations regarding unwanted short- and long-term aftereffects.

## 1 Introduction

The science of deception detection has become a steady area of research in psychology. Detecting lies and deception is not only of personal relevance in day-to-day situations but since many decades an essential part of criminal investigations and trials. Unfortunately, persons seem to be surprisingly tenacious in their assumption to catch liars by means of non-verbal signs (Bogaard and Meijers, 2022) although neither non-verbal communication nor micro expressions as a means for lie detection demonstrate sufficient reliability (DePaulo et al., 2003; Burgoon, 2018; Jordan et al., 2019) and both have been criticized as ineffective (Vrij et al., 2019). The analysis of verbal content however is a more reliable and valid approach to differentiate true from fabricated statements (Volbert and Steller, 2014; Vrij, 2014; Amado et al., 2015, 2016; Oberlader et al., 2016, 2020). Verbal content analysis examines a statement regarding a specific event for particular content criteria (Volbert and Steller, 2023). One of the most prominent tools of verbal content analysis is the Criteria-Based Content Analysis (CBCA), which builds on the finding that experience-based statements contain more individual details than fabricated accounts. In its core, it is a compilation of characteristics that mark experience-based statements (e.g., markers of autobiographical memory, such as reproduction of emotions or conversations, or script-deviant information, such as unexpected complications or external associations) as opposed to fabricated lies. CBCA was designed and is used as one analytical step in the more comprehensive method of Statement Validity Analysis (SVA), to determine the credibility of alleged victims’ statements in criminal court proceedings across various European countries (Volbert and Steller, 2014), most often regarding presumed sexual offenses. Such cases frequently lack evidence or eye witnesses, hence resulting in a statement-against-statement constellation. If the accused denies the offense, the only remaining evidence is the incriminating testimony of the alleged victim. To address this issue, courts appoint forensic psychologists to deliver an expert opinion on the credibility of the testimony. When forensic psychologists are involved as expert witnesses in criminal law proceedings, their evaluations are of central importance, because judges usually base their verdict on the psychologists’ expertise (König and Fegert, 2009; Leve et al., 2021). Regarding the high impact of CBCA in practice, the method should be ground on empirical evidence showcasing high objectivity, reliability and validity.

## 2 Traditional research on criteria-based content analysis

Based on practical experience and research on verbal criteria that was assumed to be associated with the credibility of child witnesses’ testimony in trials for sexual offences in Sweden (Trankell, 1963) and Germany (Undeutsch, 1967; Arntzen, 2011) from the 1960s onwards, CBCA was developed in the late 1980s by Steller and Köhnken (1989) as a formal list including 19 criteria. Although CBCA was introduced as a tool to verify truthful statements, in the decades to come CBCA has become the state-of-the-art method to differentiate liars from truth-tellers as well (Oberlader et al., 2016; Brennen and Magnussen, 2022; Vrij et al., 2022). While other approaches of content analysis including Reality Monitoring (Johnson and Raye, 1981; Sporer, 1997, 2004) and verifiability analysis (Nahari et al., 2014) exist, we focus on CBCA in this overview since it is common practice in German court proceedings as ruled by the German Federal Court of Justice (BGHSt 45, 164; Fiedler and Schmidt, 1999).

The criteria list by Steller and Köhnken (1989) is based on and the result of the operationalization of the hypothesis that statements about self-experienced events differ in quality from fabricated accounts (Undeutsch, 1967). On the one hand, quality refers to contextual aspects of accounts, particular the vividness of the statement. The quality of a statement is high if actions are embedded spatiotemporally in the routine of the stating person, if actions are integrated in a chain of events and if conversations are adequate for the situation described. On the other hand, quality refers to strategic aspects such as lack of memory or self-deprecation that a lying person would not include. Although these criteria were initially not derived from theoretical or empirical considerations but based on practical experience, they reflect theoretical and empirical considerations. The theory behind the CBCA assumes that (alleged) victims’ statements are cognitive tasks with different demands depending on whether the statement is truthful or fabricated (Volbert and Steller, 2014). Truth telling witnesses draw their statements from event-specific autobiographical representations encoded in the episodic memory. The representations therefore contain specific and spatiotemporally details making the verbalized experience strongly individual in character (Conway and Pleydell-Pearce, 2000). Emotional events are remembered particularly detailed, contextually integrated and vivid (Kensinger, 2007; Bowen et al., 2018; Howe et al., 2018). Liars on the other hand must construct their fabricated statement from knowledge stored in the semantic memory. The semantic memory contains general information and facts, knowledge about places, people and cognitive scripts about what is (stereo-) typical for certain situations based on prior experiences. Therefore, fabricated statements about a certain crime, for example, a sexual offense, are expected to be more schematic, vague, shallow and less individual or emotionally involved in character compared to true accounts.

Focusing on these underlying memory processes, Niehaus (2008) modified the criteria list of Steller and Köhnken (1989). The author categorized the criteria into three aspects concerning the statement in (a) general, (b) motivational and (c) Memory related characteristics. The first aspect refers to the statement in its entirety concerning essential conditions of a truthful statement as being detailed and reasoned. In reference to the described cognitive demands and the dependence on and retrieval of information from general cognitive scripts while producing a fabricated statement, non-motivational characteristics refer to the complexity and vividness of a statement. In general, a statement is high in quality if it is intertwined with features individual and non-interchangeable to the described situation in terms of storyline and involved persons. According to Niehaus et al. (2005), a lying person aims to appear competent (avoidance of insecurities, lack of memory, effort to remember and spontaneous corrections) and morally seamless (avoidance of self-accusations or doubts about one’s own testimony) to depreciate the accused in order to undermine his and emphasize one’s own credibility and to prevent doubts about one’s own statement by presenting it as inconspicuous as possible. Hence, these motivational aspects concerning strategic self-representation are likely absent in truthful statements.

This classification was extended by Volbert and Steller (2014) by further subdividing cognitive and strategy-related characteristics (see Table 1). The categorization emphasizes that different groups of content criteria might have different significance in differentiating between truthful and fabricated statements. For instance, lying persons might also strive to integrate criteria from the autobiographic episodic memory group in their statement, but might avoid integration of script-deviant details and characteristics concerning the absence of strategic-self representation.

**Table 1:** Modified version of content criteria by Volbert and Steller (2014).

Autobiographic episodic memory	Script-deviant details	Absence of strategic self-representation
*Episodic autobiographical memory:* Quantity of details Contextual embedding Description of interactions Reproduction of conversations Accounts of subjective mental state Attribution of perpetrator’s mental state	*Script-deviant/-irrelevant information:* Unexpected complications during the incident Unusual details Superfluous details Related external associations Detailed characteristic of the offense *Details not comprehended:* Accurately reported details not comprehended	*Memory-related deficits:* Unstructured production Spontaneous corrections Admitting lack of memory *Content that cast doubt on credibility:* Raising doubts about one’s own testimony *Other problematic contents:* Self-deprecation Pardoning the perpetrator

The quantity and quality of reality criteria are seen as an indicator of experience-based events. However, there is no “cut-off score” or standardized, evidence-based decision rule as to how many criteria are necessary to make a final judgement regarding the truthfulness of a statement. Steller and Köhnken (1989) as well as Volbert and Steller (2014) emphasize that the CBCA is not a simple checklist and that the criteria need to be evaluated taking personal variables (e.g., age, intellectual ability, personal narrative style) and event variables (complexity, time-interval until the interview, single vs. multiple events) into account to set a baseline of what kind of a statement can be expected. The actual statement needs to be evaluated in comparison to this expectation (“quality-competence comparison”). Similar to structured professional judgement (SPJ) methods in risk assessment of offender’ recidivism, the individual weighting of each criterion contributes to the final judgement (Hart et al., 2016). However, different operationalization and weighting of the criteria result in reduced interrater-reliability (Hauch et al., 2017). Regarding validity, meta-analyses conducted by Oberlader et al. (2016, 2020) and Amado et al. (2015, 2016) found ranging effect sizes from small, medium to large for CBCA. In their first meta-analysis Oberlader et al. (2016) found that with equally high sensitivity and specificity, the effect size resulted in a hit rate of almost 70% and a false alarm rate of 30%. Nevertheless, in their second meta-analysis, correcting for research bias, Oberlader et al. (2020) conclude, that *“CBCA works more or less well”* (p. 401). The meta-analysis found no significant moderating effect of the participants’ age, which implies that the method should work for child and adult witnesses similarly. Oberlader et al. (2016) describe two traditional approaches that are currently being used to investigate the validity of CBCA: *field studies* that investigate statements from alleged victims in criminal court proceedings (e.g., Roma et al., 2011) and *laboratory studies* that analyze truthful and fabricated statements that participants were instructed to create in experimental settings (e.g., Vrij et al., 2004). Both traditional methods come with certain disadvantages. Generally, field studies have the shortcoming of decreased internal validity because of a high variety in the assessment procedure or classification of a case as true or fabricated, for instance on the basis of confessions to the police, SVA results or judicial convictions. Nonetheless, field studies in CBCA research have to assume “ground truth” about whether the included statements of alleged victims were indeed true or fabricated. However, even in the case of a credibility assessment or a judicial conviction this cannot be established for certain. The judicial decision might also be influenced by the experience of the judge or the self-confident appearance and persuasiveness of the evaluator in the court room who conducted the CBCA. Additionally, field studies lack standardization, and the aspect of interrater reliability is usually not being addressed (Krahé and Kundrotas, 1992). On the other side, laboratory studies come with the disadvantage of decreased ecological validity. Some traditional CBCA research designs use participants self-reported events from their past, either truthful or fabricated. Other laboratory studies stage an event for participants to experience and question them about it later on (e.g., Vrij et al., 2004). In former research, events ranging from taking part in a photography session (Akehurst et al., 2001) to being part of or watching a staged or videotaped theft (e.g., Porter and Yuille, 1996; Vrij et al., 2000) or robbery scenario (e.g., Bensi et al., 2009) were used in adult samples. Both laboratory study types come with disadvantages: The “ground truth” of experienced events from the participants’ past cannot be established for certain, even when using external confirmation to verify the participants’ statements (e.g., Santtila et al., 2000). Staged events will never reach an emotional response similar to an actual (sexual) victimization. Oberlader et al. (2020) found larger effect sizes for the CBCA field studies than for CBCA laboratory studies. The same was reported by Hauch et al. (2017) regarding their meta-analysis of reliability. However, most field studies include child victims’ statements, while laboratory studies usually include statements from grown-up students.

To sum up, the challenge in validating content-based techniques such as CBCA is to design studies that can experimentally control “ground truth,” as known from laboratory studies, but also increase the ecological validity of staged events so that they more closely resemble the crime scenarios, especially sexual assault, for which CBCA is usually applied in practice. Many authors discuss the ethical limitation of creating scenarios resembling the experience of a sexual victimization in the laboratory (Steller and Köhnken, 1989; Krahé and Kundrotas, 1992; Oberlader et al., 2020). Oberlader et al. (2016) demand that “*content-based technique research conditions that are as realistic as possible are still mandatory, as invalid results have serious consequences in real-life settings*” (p. 453).

## 3 Is virtual reality the new game-changer?

Virtual reality (VR) is a three-dimensional artificial environment that is experienced through sensory stimuli (such as sights and sounds) provided by a computer and which enables a real-time interaction. Numerous studies from different areas of research have already used VR techniques including military, economical, rehabilitation, education but also memory research studies (for a review see Smith, 2019). VR scenarios differ in terms of *immersion* and *presence*. *Immersion* is classified as the level to which the VR system produces a naturalistic portrayal of the sensory elements of a specific virtual environment (Smith, 2019). Immersion depends mostly on technical parameters such as visual details or visual fidelity when the head can be turned. A higher degree of immersion enables the study participant to truly dive (or *immerse*) into the virtual scenario and is found to increase memory performance and memory retrieval (e.g., Gamberini, 2000; Ruddle et al., 2011; Wallet et al., 2011; Harman et al., 2017; Dehn et al., 2018; LaFortune and Macuga, 2018; Schöne et al., 2019; Kisker et al., 2021). *Presence* on the other hand describes the degree of “the feeling of being there” (Nash et al., 2000), i.e., the subjective feeling of attending the virtual environment and forgetting about the real surroundings (e.g., a study laboratory). Presence is primarily a result of the immersion degree. In his review Smith (2019) describes that increased presence in VR is positively associated with attentional selection and engagement which may increase memory retrieval.

The use of VR scenarios in research enables scientists to solve the dilemma of deciding between ecological validity and the control of the “ground truth,” increasing external and internal validity simultaneously. It can provide a balance between experimental control and the need for a more naturalistic experience (Parsons, 2015). Regarding research on the validity of verbal content analysis, using a standardized virtual scenario would enable full control over the “ground truth” of a statement about an event that was experienced in VR. The highly standardized VR material would enable a comparison of participants’ statements with the VR input, so that even small deviations from the actual VR experience can be assessed. At the same time, using VR scenarios can increase the ecological validity of CBCA research, as opposed to traditional methods (e.g., reading a case vignette, imagining an event or watching a 2D video), on various domains: (1) the mock events can be designed to resemble the criminal events for which CBCA is used in the field (e.g., sexual assault). (2) the three-dimensional and multi-sensory experience in VR increases the number of details that can be perceived and potentially included into a statement about the event. (3) using scenarios with high degree of presence and immersion should increase the emotional involvement into a situation, improve memory encoding and enable participants to include emotional details into the statement about the event.

Prior research supports these claims. For example, in a recent study on psychophysiological effects of VR, Schöne et al. (2023a) demonstrate that an immersive VR experience of height exposure in a reproduced fire-truck basket produce a similarly high emotional response (arousal and anxiety) as a real-life experience in an actual fire-truck basket and a significantly higher emotional response than a 2D video exposure of the same event. Three days after the experience, the real-life and VR group participants still remembered the ride as similarly emotional and differed significantly from the participants watching the scene on a 2D screen. While the study design is still not comparable with an emotional response activated in (sexual) victimization, Schöne et al. (2023a) demonstrate that VR scenarios are more suitable to cause high emotional response than 2D videos. Thus, immersive VR experiences that trigger such emotional responses should be encoded more strongly in the autobiographical memory, making memory retrieval and corresponding statements more richly detailed, similar to real experiences. This should create a stronger deviation in the quality of statements between VR experienced and fabricated events compared to traditional laboratory methods. Thus, compared to traditional laboratory settings, an increase of CBCA criteria can be assumed in VR settings compared to traditional laboratory settings. Prior research results support this claim, because participants demonstrated better memory performance after VR experiences in comparison to traditional input of material (Smith, 2019).

### 3.1 VR-studies in forensic research

Forensic psychological research has discovered VR scenarios for different areas of research (for an overview see Renaud et al., 2009, 2014; Fromberger et al., 2014, 2018; Romeo et al., 2019; Barbe et al., 2020; Cornet and Van Gelder, 2020; Gewehr et al., 2022; Terbeck et al., 2022; van Gelder et al., 2022). Most of these studies can be placed within the area of offending research, for instance measuring (or enhance) emotional or sexual responses and empathy in offenders, others such as Ventura et al. (2021) or Neyret et al. (2020) used VR scenarios to assess empathy with female victims of sexual harassment in community men. In the area of victim-based research or witness interviewing, building on initial findings of Pompedda et al. (2014, 2017), the “ViContact” project presents a computer-generated VR environment for teachers training them how to talk to children (avatars) about suspicions of sexual abuse (Gewehr et al., 2022). The authors are unaware of studies from the field of forensic science that used VR scenarios to compare experienced-based statements and fabricated statements using CBCA. Segovia and Bailensen (2009) came close to the area of SVA research by investigating the use of VR to (successfully) induce false memories in children. Romeo et al. (2019) used a computer-generated VR scenario in deception detection research to assess the impact of lying about a traumatic virtual experience on memory. A sample of 94 adults watched an airplane crash with dead bodies scattered on the ground including specific visual and auditory details. Participants were not personally involved in the VR scene. Unfortunately, the authors decided against a semi-structured interview that might have enabled a content-based analysis but chose different memory tasks all requiring simple “yes/no” answers to questions about details either included or not included in the VR scenario. Although the truth telling group correctly remembered significantly more details from the VR scene than participants who were instructed to deny details seen in the scenario, no control group of fabricated accounts were included in the analysis. Since the stimulus (air-plane crash) lacked self-relevance, the authors recommend to use of VR stimulus sets that are not only realistic but in which the event is self-relevant to the participants (for instance a VR scene in which participants are being attacked).

Although victim-related VR studies in the context of SVA are missing in forensic research, existing studies support the applicability of virtual scenarios to the context of victimology. Loranger and Bouchard (2017) developed a VR study for female victims of sexual assault for therapeutic purposes. They aimed to determine whether a VR immersion in an environment leading to sexual assault would trigger anxiety. A rather small sample of 30 participants were presented with a computer-generated neutral nighttime bar scenario and a following sexual assault scenario at a bus stop nearby. The sexual assault scenario featured a male avatar who sits next to the avatar of the female study participant, puts his arm around her shoulders, fondles her thigh and chest and then becomes angry and shoves her. The following actual sexual assault is witnessed via sound and blurry images. The authors found significantly more anxiety and negative feelings regarding the VR sexual assault scenario compared to a neutral bar scenario. This supports the idea that sexually threatening VR stimuli are suitable to trigger strong emotional response, although the authors did not assess whether the scenario triggered emotional responses specifically associated with autobiographic memories. Jouriles et al. (2009, 2011) validated a virtual role play including a sample of 61 (2009) and 48 (2011) women with the aim to help female students resist sexual attacks. In the 2011 study, female participants were instructed that they met the virtual male at a party and went up with him to their room to get to know each other better. The male was represented by an avatar whose behavior was controlled by a male actor actually sitting next to the female while she watched the scene in VR. The virtual scene included four stages of escalation: (1) getting acquainted, (2) mild sexual advances, (3) escalating sexual advances and (4) hostile sexual advances. Since physical touch is not possible in VR, the escalation of sexual advances was expressed verbally by the male actor. Participants in the experimental VR group demonstrated significantly more negative affect in comparison to a traditional (non-virtual) role-play. This indicates stronger emotional involvement and presence activated by a VR stimulus compared to a staged event.

**Figure 1:**
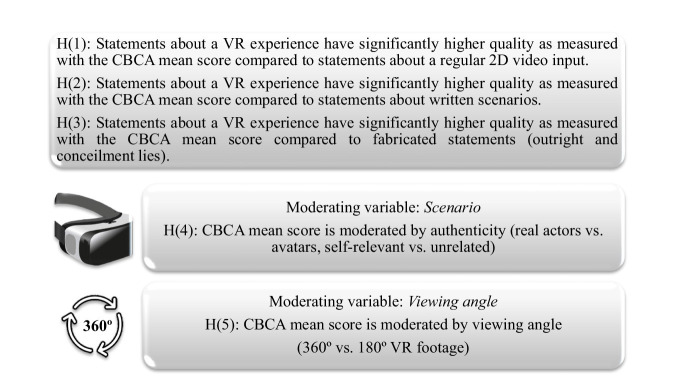
Proposal of study hypotheses.

While VR stimuli can only provide an approximation to a real-life sexually threatening situation, these studies give a first impression on the research possibilities of VR scenarios for verbal content analysis methods. They illustrate limitations future research has to address. Using avatars in VR scenes or solely letting participants watch a scene from a mere witness perspective might cause a lack of self-relevance (see Romeo et al., 2019). Hence, the scene might not feel sufficiently real, behavioral and emotional responses might not be as realistic and authentic as hypothesized. Barbe et al. (2020) state that the sense of presence increases with a convincing VR scene. Hence, using real persons (e.g., actors) instead of avatars and integrating participants in actions or conversations of the virtual scene should increase the feeling of presence and thereby the perception of the scene as real. This enables participants to experience emotional and behavioral reactions which would also emerge in real-life situations (Alsina-Jurnet et al., 2011) and thereby increase memory retrieval (Smith, 2019). Drawing from these assumptions as well as prior research, Figure 1 presents a proposal of hypotheses for future study purposes. However, getting access to suitable VR material for CBCA research is challenging. Producing a 360° VR Video with professional actors is high in effort, time and money. Initiatives such as demonstrated by Schöne et al. (2023b) or Li et al. (2017) to develop an open-source database for immersive VR 360° videos are very encouraging and will hopefully produce imitations. Freely accessible VR videos can also be found online at popular video sharing websites. Developing immersive VR videos with sexual content is even more difficult though. In our opinion, former studies described above are an encouraging groundwork to further discover the possibilities within the realm of VR regarding sexually victimizing experiences. The limitations of traditional research in the field of CBCA research call for using convincing, self-relevant VR scenarios corresponding to real-life situations to further investigate the differences between truthful and fabricated statements. In the light of the ongoing technical development in the field of virtual and augmented reality, including physically experiencing scenarios first-handed, the options for validating content-based techniques are extensive. For instance, prior research also used VR pornography for scientific purposes (for a review see Evans, 2023). VR pornography could be used to assess a “base rate” regarding adults’ reports of sexual encounters in general. Empirical evidence regarding descriptions of consensual sexual encounters could be valuable when evaluating adults’ statements of alleged sexual offenses in practice, for instance in cases of interpersonal sexual violence. Milani et al. (2022) already demonstrated the feasibility and value of using “Virtual Reality Erotica” with female participants, but also emphasize ethical limitations.

## 4 Ethical considerations of VR usage for validating content-based methods

As shown above, VR experiences can lead to emotional and physical response. Different studies also found empathetic response and stress after the VR exposure (for an overview see Sora-Domenjó, 2022). Therefore, ethical considerations are necessary when discussing using VR stimuli for validating content-based methods. In general, VR experiences differ from traditional stimuli in terms of (a) the appearance of motion (cyber) sickness, (b) potential information overload, (c) intensification of emotional response in a VR environment and (d) cognitive, emotional or behavioral insecurities after re-entering the “real world” (Behr et al., 2005). Mandary and Metzinger (2016) underline the importance of cautiously designing VR studies and advise considering duration, content, screening procedure, false sense of agency, explicit consent of data privacy and long-term immersion in social VR environments. The authors argue in favor of the development of an empirically based standard set of exclusion criteria, particularly taking existing psychological disorders (e.g., psychosis, bipolar disorder, suicide risk; see Rothbaum et al., 2014) and undetected psychiatric vulnerabilities into account (i.e., Depersonalisation/Derealisation Disorder; see American Psychiatric Association, 2013). Furthermore, it is emphasized that taking care of participants before, during and after a VR experience is central to monitor emotional response. Loranger and Bouchard (2017), whose study on validating a virtual environment for sexual assault victims was approved by an ethics committee, claimed that the experiment was well tolerated by the women and no one needed post experimental support. Prior to immersion, participants were informed that they could end the immersion at any time. Furthermore, the authors asked for additional verbal consent from the participants before progressing to the next stage of escalation. The research regarding a virtual roleplay conducted by Jouriles et al. (2009) was also approved by an institutional (ethical) review board despite including female participants with personal experiences of being sexually assaulted. A post-experience counseling was offered in case of negative aftereffects (however, whether participants accepted the offer was not reported). Neyret et al. (2020) addressed potential post-experience negative aftereffects by presenting a second VR experience in which the female avatar that was sexually harassed in the main VR input stated that she was well and had experienced no pain. Neyret et al. (2020) reported an observed emotional relief in most of the participants which was not assessed standardized though.

However, when discussing the use of VR to create (sexual) harassment scenarios to validate content-based techniques, the risk of long-term effects is not sufficiently discussed. Studies involving sexual assault VR scenarios need to take potential triggering events in the participants sexual history into account, for instance prior experiences of sexual victimization. Therefore, sexual assault experiences could serve as an exclusion criterion, or participants need to be instructed and watched carefully before, during and after a VR experience as described above. If study participants are being sexually harassed or otherwise victimized in the real world following the VR experience, potential consequences should be anticipated. For instance, although the VR experience was tolerated well, it might increase emotional and mental load and reduce resilience for future stressful situations. Thus, future stressful situations might not be coped with as well as without the VR experience or even serve as a trigger to retrieve memories of the VR harassment experience that – as discussed above – has the potential to be remembered as equally emotional exciting as real-time events. Slater et al. (2020) also emphasize: “*We change through our experiences: experiences produce changes in the body and the brain. In other words, just as real-life experiences have after-effects, so virtual experiences may have physical, emotional, and cognitive after-effects which may be beneficial or harmful*” (p. 8). Debating “worst-case ethical problems,” Slater et al. (2020) likewise discuss whether participants might come to remember the VR events as if they had been real, and fail to distinguish events that really happened and those that happened in VR over time. Although these scenarios might be “worst case” discussion, researchers thinking of using VR to create harassment exposure should consider longer follow-up periods to assess post-experience aftereffects.

## 5 Conclusion

Prior research supports the idea of using immersive virtual reality scenarios for validating verbal content analysis methods like CBCA. Since the prize for high-quality VR hardware is constantly reducing, using VR stimuli is not necessarily more costly than traditional research paradigm and it comes with the advantage of enhanced ecological validity. The utility and ethical justifiability of VR scenarios to assess women’s reactions in victimizing situations have been demonstrated. Considering these advantages, we argue that using VR scenarios in a highly controlled laboratory has the potential to solve the ongoing ethical dilemma of validating content-based techniques. Following our argumentation, in theory, study participants experiencing some sort of victimization in immersive virtual environments should be able to produce a high-quality statement including emotional and situational details as well as personal involvement that could be analyzed with content-based techniques such as CBCA. Beyond, using a standardized VR environment comes with the advantage of maximum control over the event by knowing the “ground truth” of the presented situation. However, since VR can serve as a stimulus which evokes a feeling of reality, ethical considerations must be taken into account. Although we chose to limit our discussion of the utility of VR scenarios for studies on adults only, many problems in validating content-based techniques concern adult and child witnesses’ statements alike. This must be addressed by future research in this field. By discussing the advantages and disadvantages of using VR in forensic research, we aimed to add an impulse to the present traditional efforts of validation. Adding VR stimuli to the methodological toolbox, innovative studies may be conducted to unfold further empirical limitations of the CBCA, for instance regarding interrater-reliability, objectivity and standardization, testifying motivation, different base rates etc. Since the CBCA is usually used in criminal court proceedings, experts should be enabled to produce replicable results. Stronger empirical evidence for this “best practice” method helps practitioners to make their final evaluation transparent to decision makers and alleged victims alike.

## Author contributions

JAI: Conceptualization, Writing – original draft, Writing – review & editing. TV: Conceptualization, Writing – review & editing. SG-K: Conceptualization, Supervision, Writing – review & editing.
